# Protein interface classification by evolutionary analysis

**DOI:** 10.1186/1471-2105-13-334

**Published:** 2012-12-22

**Authors:** Jose M Duarte, Adam Srebniak, Martin A Schärer, Guido Capitani

**Affiliations:** 1 Paul Scherrer Institut, Villigen, CH-5232, Switzerland; 2 SyBIT, ETH Zurich, Zurich, Switzerland; 3 Present address: Institute of Molecular Biology and Biophysics, ETH Zurich, Zurich, CH-8093, Switzerland

**Keywords:** Protein structure, Protein-protein interfaces, Crystal interfaces, Classification, Evolutionary, Core residues, Web server

## Abstract

**Background:**

Distinguishing biologically relevant interfaces from lattice contacts in protein crystals is a fundamental problem in structural biology. Despite efforts towards the computational prediction of interface character, many issues are still unresolved.

**Results:**

We present here a protein-protein interface classifier that relies on evolutionary data to detect the biological character of interfaces. The classifier uses a simple geometric measure, number of core residues, and two evolutionary indicators based on the sequence entropy of homolog sequences. Both aim at detecting differential selection pressure between interface core and rim or rest of surface. The core residues, defined as fully buried residues (>95% burial), appear to be fundamental determinants of biological interfaces: their number is in itself a powerful discriminator of interface character and together with the evolutionary measures it is able to clearly distinguish evolved biological contacts from crystal ones. We demonstrate that this definition of core residues leads to distinctively better results than earlier definitions from the literature. The stringent selection and quality filtering of structural and sequence data was key to the success of the method. Most importantly we demonstrate that a more conservative selection of homolog sequences - with relatively high sequence identities to the query - is able to produce a clearer signal than previous attempts.

**Conclusions:**

An evolutionary approach like the one presented here is key to the advancement of the field, which so far was missing an effective method exploiting the evolutionary character of protein interfaces. Its coverage and performance will only improve over time thanks to the incessant growth of sequence databases. Currently our method reaches an accuracy of 89% in classifying interfaces of the Ponstingl 2003 datasets and it lends itself to a variety of useful applications in structural biology and bioinformatics. We made the corresponding software implementation available to the community as an easy-to-use graphical web interface at http://www.eppic-web.org.

## Background

Protein crystal lattices contain two kinds of interfaces: biological ones (as present in physiological conditions) and crystal packing ones (non-specific), indistinguishable by crystallographic means. Traditionally they have been assigned by visual inspection alone, but their identification has increasingly become a challenge due to the sheer complexity of the macromolecular objects that modern structural biology tackles nowadays. A series of breakthroughs in protein production and structure determination techniques, especially in protein crystallography, nuclear magnetic resonance and electron microscopy, have enabled researchers to solve the structure of macromolecular complexes and oligomeric proteins of very large size, sometimes composed of many copies of different kinds of subunits. Prominent examples in this respect are for instance fatty acid synthase [1] and the recently solved immunoproteasome [2]. Another important trend is the increasing automation of the structure determination pipeline through structural genomics efforts, often producing protein structures before thorough biochemical characterization. Reliable computational tools are thus needed to decide which interfaces are the biologically relevant ones and consequently what is the biological assembly in the crystal. The need for such tools is not limited to crystallography: integrated approaches merging electron microscopy, proteomics and crystallography are being employed to tackle very complex entities such as the nuclear pore complex [3,4]: there, researchers determine the structures of individual components in order to fit them into a lower resolution global electron density map derived from electron microscopy data. It is vital, in order to obtain a correct fit, to know if the assemblies of the components obtained by crystallography are biologically relevant.

In the last fifteen years several computational methods have been developed to distinguish biological interfaces from crystal contacts. The first of them relied on interface area analysis [5] and was followed by approaches based on sequence conservation [6-8], combination of geometrical and other properties such as conservation via machine learning [9-11] and thermodynamic estimation of interface stability [12]. This last method, implemented in the PISA server, proved to be the most successful and is the current *de facto* standard in the field. An interesting approach, PROTCID [13,14], infers information about the biological significance of interfaces from their presence in multiple crystal forms of the same protein (if available).

In this article we present an integrated approach to the problem that relies on evolutionary analysis of the interfaces and on a novel geometric criterion. In a previous, proof-of-concept work [15] we employed *Ka/Ks* ratios as the evolutionary metrics for the selection pressure acting on protein-protein interfaces in crystals. *Ka/Ks* ratios are a well-established tool in the field of molecular evolution: they measure the ratio of the number of non-synonymous substitutions per non-synonymous site to the number of synonymous substitutions per synonymous site in a multiple alignment of coding sequences [16]. We compared the *Ka/Ks* ratio averages of interface rim and core sets to detect if the selection pressure acting on core residues was significantly stronger than that of rim residues. This approach had three main limitations: first, its recall was limited since in many cases not enough homologs could be found to run a significant *Ka/Ks* ratio estimation. Second, *Ka/Ks* value estimation was slow, bringing the duration of most runs up to several hours. Third, no easy-to-use public implementation was available.

Our new approach, named EPPIC (Evolutionary Protein-Protein Interface Classifier), overcomes all the above limitations, introduces two novel criteria for detecting biological contacts and, most importantly, achieves a very high level of accuracy. Additionally we implemented it in a robust freely available software package and offer it to the community in an easy-to-use graphical web interface.

## Results and discussion

EPPIC is an approach for distinguishing biological interfaces from lattice contacts in crystal structures using evolutionary information from protein sequences. Some early attempts [7,8] in this direction, using sequence entropies as metrics for selection pressure, did not achieve levels of accuracy high enough to make them competitive with methods like PISA, which estimates the thermodynamic stability of an interface to predict whether it should exist in solution (biological interface) or only in the crystalline state (crystal contact). To date, PISA is the *de facto* standard to address the biological interface versus crystal contact issue and to predict the biologically relevant assembly of protein structures. Since PISA makes no use of sequence information, complementary methods that employ the wealth of sequence data available are particularly needed, especially as the size of biological sequence databases has increased exponentially in the last years and will only keep increasing further in the near future.

Our recent approach [15] aimed at demonstrating the feasibility of an evolution-based method measuring interface selection pressure at the coding-sequence level. Having achieved that goal, we set out to develop a completely new, more powerful and general approach to the problem, overcoming the limitations described in the introduction. First of all, we introduced a new geometric analysis criterion, based on the number of core residues in an interface, which represents by itself a powerful predictor of interface character. This allows us to formulate an interface assignment even when not enough homologs to the query are available for evolutionary analysis. Second, we have re-evaluated the use of sequence entropies instead of *Ka/Ks* ratios as a metrics for selection pressure. We found out that, with stringent criteria for homolog selection, better redundancy reduction of sequences and thanks to the increasing amount of sequences currently available, we could reach a better performance than that achieved with *Ka/Ks* ratios. The usage of entropies brings the advantage of making calculations much faster but also of simplifying the computational workflow. Third, we have introduced a new way to exploit the difference in selection pressure between interface and surface residues. Comparing the average sequence entropies of interface and non-interface residues is an approach pioneered by Elcock & McCammon [7]. That early attempt, however, was limited by the small size of sequence databases at the time and most importantly by biasing factors acting on surface residues, *e.g.* allosteric binding sites, unknown interfaces to other partners, external active sites and the like. We modified that approach substantially, first of all by comparing only interface core residues with surface residues, and by introducing a random pooling of surface residues that enables us to compute more statistically robust scores of selection pressure acting on the interface core residues with respect to the surface “baseline”. A similar surface sampling approach was also used successfully by Valdar and Thornton [17] in order to analyze conservation in a small set of homodimer interfaces.

The above three criteria, combined with further statistical considerations on interface area, allow us to achieve a performance of 89% accuracy on a minimally modified version of the Ponstingl 2003 dataset [18] as compared with 84% accuracy achieved by PISA on the same interfaces.

### Compilation and annotation of new reference datasets

An important issue we tackled in this study was that of reference datasets of crystal contacts and biological interfaces. We identified this as one of the most important issues in the computational prediction of interface character and believe this particular problem has not received enough attention in previous studies. Experimental methods for oligomeric state determination are themselves prone to artifacts and it is rather common in the literature to find debated assignments, based on contradictory experimental data. It is thus essential that the data to be used for method developing and benchmarking have 100% clear experimental backing. The crystallographic accuracy of the structures is also vital: we realized that some of the most frequently used datasets in the literature contained some structures not following the most stringent crystallographic quality criteria, since they were solved many years ago and predated the use of quality measures such as the free R-factor [19].

Another important issue that has been mostly neglected is the distribution of areas of the interfaces used to train or benchmark classifier algorithms. As demonstrated already by Janin [5], an exponential decay relationship exists in the distribution of areas of crystal interfaces: the bulk of the crystal interfaces known to date have areas below 1000 Å^2^ with very few representatives above that value. It is also well known that biological interfaces on the contrary tend to exhibit large areas [20], with a majority of cases from 1000 Å^2^ and above. An overlap region exists where both kind of interfaces are frequent in the area values of approximately 800 Å^2^ to 2000 Å^2^. Thus an interface-classifying method should always take this into account and use this area distribution as a baseline for predictions. As Ponstingl [21] already noted, a simple classifier based on area alone achieved high accuracy in interface assignment. In introducing our own reference datasets we prioritized having a distribution of areas that is out of the trivially classifiable region. This issue was first recognized and partly addressed in the work of Bahadur *et al*. [22], where they included a crystal interface in their dataset only if its total buried area was above 400 Å^2^.

**Figure 1:**
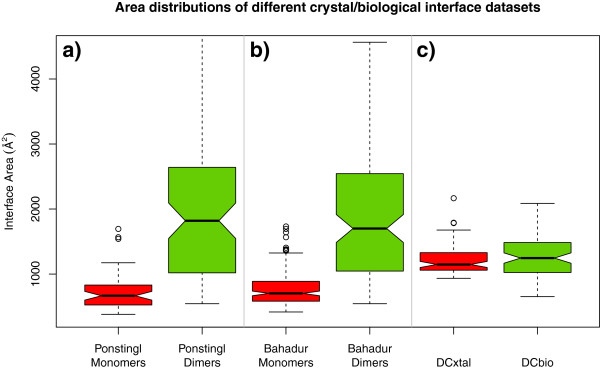
**Distribution of interface areas in benchmarking datasets: Boxplots for a) Ponstingl Monomers in red and Ponstingl Dimers in green, b) Bahadur Monomers in red and Dimers in green c) DCxtal in red and DCbio in green.** Our datasets focus on the range of areas where the two types of interfaces overlap the most, making it most difficult to predict their character.

We thus created our own reference datasets, adopting a three-fold strategy: 1) only use entries for which the oligomeric structure is clearly experimentally verified 2) include only crystal entries that fulfill a series of quality check criteria (see Methods), 3) focus on the range of interface areas where it is really difficult to distinguish crystal from biological contacts. We compiled two Duarte-Capitani datasets: one of large crystal contacts (DCxtal), the other of small biological interfaces (DCbio). DCxtal contains 78 entries validated as monomers, with 82 crystal interfaces of at least 1000 Å^2^. For comparison, in the Bahadur set the lower limit for crystal interface area was set at 400 Å^2^. Surely the growth in the number and average quality of available crystal structures has made the compilation of a sizeable dataset of large crystal contacts easier than in the past. DCbio consists of 74 oligomers, with 83 validated biological interfaces. Both datasets are listed in detail in Additional file 1: Tables S1 and S2, respectively. In Figure 1 we plotted the area distribution of the entries in our datasets and two others for comparison: the Ponstingl 2003 dataset of monomers and dimers [18] and the Bahadur homodimer and monomer datasets [22]. The boxplots show clearly very different area distributions, being our datasets a mixture of biological and crystal interfaces belonging exclusively to the overlapping area region. We also compared the DC sets with the PiQSi database [23]: while only 23 DC entries (out of 152) were present in PiQSi, their assignments were 100% in agreement with the PiQSi ones.

### Geometry criterion: core size

**Figure 2:**
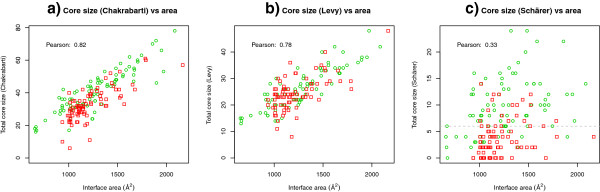
**Correlation of core size in different definitions to area.** Dots represent interfaces of the DCbio (green circles) and DCxtal (red squares) datasets. The first two definitions show high correlation, whilst the definition of Schärer, used in this work, has a much lower correlation. In the third plot we marked the core size value of 6 (cut-off used for the geometry classifier) with a horizontal line.

The idea of dividing the residues of the interface, *i.e.* those that bury some surface area, into different classes appeared early in the protein interface literature. LoConte *et al.*[24] proposed a first classification based on atoms rather than residues, dividing them into 3 classes which they called A, B and C. The fully buried atoms formed class B, while classes A and C were subdivisions of the partially buried ones. Later Chakrabarti and Janin [25] introduced the concept of core residues as those residues having at least one fully buried atom. This definition was later used by Guharoy & Chakrabarti in their pioneering work on the relative average entropies of core and rim residues in interfaces [8]. Schärer *et al.*[15] substantially modified the definition of core residue, basing it on the percentage of the accessible surface area (ASA) that becomes buried upon interface formation. The cut-off for defining a residue as core was set by Schärer *et al.* at 95% burial (BSA/ASA). Levy [26] used a more complex scheme with 3 categories: core, rim and support. The scheme uses, as well as BSA and ASA, the relative surface accessibilities (rASA), i.e. the ASA of a residue X relative to its ASA in a reference extended tripeptide GLY-X-GLY. In Levy's definition a residue is “core” if: 1) its rASA in the monomer is larger than 25% and 2) its rASA in the complex is smaller than 25% (Table 1). The Schärer definition proved effective when employed to divide interfaces into a rim and a core set, the average *Ka/Ks* ratios of which were then compared to classify the interfaces into “crystal” or “bio”. As part of the present work we analyzed the predictive power of the three residue-based core definitions (Chakrabarti, Levy, Schärer) when using the number of core residues as a simple geometric criterion to categorize interfaces as biological or crystal contacts. Figure 2 displays the number of core residues (from now on called core size) found in our datasets of biological interfaces and crystal contacts by using the Chakrabarti, Levy and Schärer core definitions, respectively. The core size of each interface is plotted versus its area. Notably, while the two former core definitions lead to a quite strong correlation of core size with interface area (Pearson correlation coefficients 82% and 78%), the latter is much less correlated (Pearson 33%). Moreover in many cases it seems to clearly separate crystal from biological interfaces. For our two datasets it is able to tell bio interfaces apart from crystal interfaces with 80% sensitivity and 73% specificity, which makes it *per se* a powerful discriminator of interface character. In their 2004 work Bahadur *et al*. [22] presented two geometric parameters that were also very good at discriminating interfaces, namely the fraction of buried atoms and the non-polar interface area. It must again be underlined that the data used in that study was very different: their crystal interface areas were above 400 Å^2^ whilst here our DCxtal interfaces are above 1000 Å^2^.

**Table 1:** Interface core definitions from the literature

	***Chakrabarti***	***Levy***	***Schärer***
Core definitions	Residues with at least 1 atom with BSA/ASA(u)=1	Residues with rASA(u)>0.25 & rASA(c)<0.25	Residues with BSA/ASA(u)>0.95
Example bio interface: [PDB:1N8P] interface 1 (total BSA=1969 Å^2^)	26+27	24+24	4+3

Interface residues are those for which BSA>0, core residues are then a subset of those. The values of core residue sizes for a typical biological interface example chosen from one of the entries in DCbio are shown (the 2 numbers corresponding to first and second partner of the interface). BSA is defined as BSA=ASA(u)-ASA(c), relative ASA as rASA=ASA/ASA(GLY-X-GLY). *u* and *c* stand for uncomplexed and complexed respectively.

**Figure 3:**
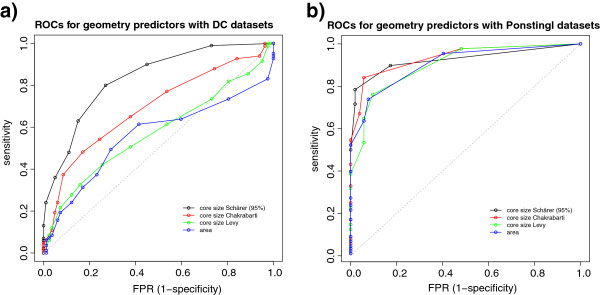
**ROCs for different geometric indicators.** The ROC curves represent the predictive power of different geometric parameters: core size (Schärer’s definition), core size (Chakrabarti’s definition), core size (Levy’s definition) and total buried surface area. In panel a) the datasets used are our DCbio/DCxtal, whilst in panel b) the Ponstingl datasets were used. Not much difference can be appreciated if using Ponstingl’s dataset, since it contains interfaces that are too clearly separable by area. When we use the DC datasets, it becomes apparent that Schärer’s core definition exhibits superior performance compared to the other geometric indicators.

As another way of displaying the predicting power of Schärer's core definition we produced ROC curves (Figure 3) depicting the ability of various geometrical parameters to predict the character of a) our DC bio/crystal interfaces and b) Ponstingl’s bio/crystal ones. Schärer's definition outperforms the others and also the interface area as predictors. This difference only becomes apparent when using the datasets that focus on the difficult to predict region (a). If we use a more conventional dataset with a typical area distribution (b) the difference does not appear. This is striking as previous studies [9,27] of several geometrical interface parameters, including some based in Voronoi tessellation, found that area ranked first in prediction power compared to the other parameters.

**Figure 4:**
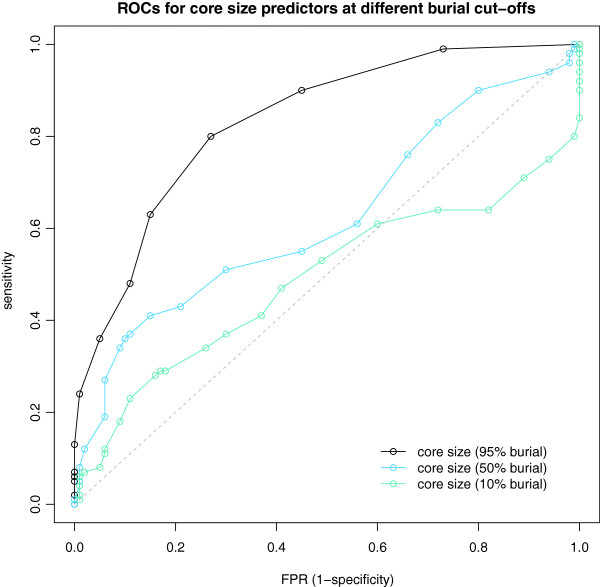
**Schärer’s core definition at different cut-offs.** ROC curves for Schärer’s core size at different BSA/ASA cut-offs as predictor for the DC datasets. The 95% burial cut-off has a clear advantage over the lower cut-off core definitions.

Schärer's definition uses a percent burial cut-off to assign residues to core, so the question arises as to what an optimal value for this cut-off in terms of interface classification is. Strikingly, the 95% cut-off appears much more powerful than lower ones. We plot in Figure 4 the ROC curves of the core size at different cut-offs (95%, 50% and 10%) as predictors of interface character for our DC datasets. It is apparent that when one includes more and more partially buried residues the predictive power decays rapidly.

The core residues thus defined seem to be an essential interface determinant. Interestingly the definition is in agreement with that of hot spot residues introduced by Bogan *et al.*[28] and offers a possible explanation as to why the number of core residues is so powerful in distinguishing biological interfaces. In that study, the authors compiled a set of site-directed mutagenesis studies on interface residues and found that only a few well-buried residues contributed the most to the binding energy of the interface. Moreover, all residues that contributed significantly to the binding energy were fully (or nearly fully) buried, whilst partially buried residues were never found to significantly contribute to the energy. Thus, full burial was a necessary but not sufficient condition for a residue to be a hot spot.

It must also be noted that crystallographic accuracy is essential for the effectiveness of the geometry criterion, the full power of which can only be seen when using sets of good quality crystal protein structures. A striking example is the structure of bovine interferon gamma, solved first at 3 Å resolution ([PDB:1RFB]) and later again at 2 Å resolution ([PDB:1D9C]) in the same crystal form. The area of the dimer interface changes from 2600 to 3600 Å^2^ from the first to the second, but more importantly the number of core residues leaps from 1 in the first case to 36 in the second.

### Estimation of selection pressure: sequence entropies

As mentioned above, in this work we decided to move from the *Ka/Ks* ratio selection pressure metrics to sequence entropies at the amino-acid level. We realized that we could see very good differential selection pressure signal at the interfaces by carefully choosing the homolog sequences to measure the entropies. Most importantly we decided to be very conservative in the amount of homologs to use, cutting the homolog list at a sequence identity value as high as 60% (extending to a hard cut-off of 50% when not enough homologs are found). There are mainly two reasons for this choice.

First, by staying in the very high identity region we avoid the risk of introducing errors in the alignments and we can rely on the assumption that the structures of homologs used in the alignment are very well conserved. From knowledge gathered over the years of CASP structure prediction experiments, it is known that alignment accuracy is very good only down to ~50% sequence identity, medium to good in the 30-50% identity region and low below 30% identity (the “twilight zone”) [29,30]. These assessments done over the different CASP experiments are based on the gold-standard of a structural alignment to the best template [29].

As a second point the quaternary structure of proteins and thus interfaces seem to be less conserved than that of the tertiary structure. Poupon and Janin [31] estimate that 40% is a reasonable limit to the reliability of a good quaternary structure homology, thus it seems dangerous to consider sequence homologs below that 40% level.

Strikingly, almost all methods for interface classification or prediction until now have used much lower sequence identities for measuring conservation. For instance a few studies [7,8,32,33] used the well-known HSSP database to get their alignments. HSSP uses 25% as the identity cut-off for sequences with length above 80 residues (a majority of PDB proteins these days) [34]. Valdar *et al.*[6] select their homologs by performing a maximum of 20 PSI-blast iterations with an inclusion e-value cut-off of 10^-40^ which results in identities as low as 5%. Caffrey *et al.*[35] even compared two types of alignments: a “diverse” one, aimed at capturing paralogs, and a “close” one to contain only orthologs. The former had a very generous homolog inclusion cut-off (blast with e-value cut-off of 0.001) while the latter took close orthologs from selected species in the same taxonomic kingdom. This second type of alignment, although more stringent, is not comparable to those computed here, as it typically contained very few sequences (10 to 15).

**Figure 5:**
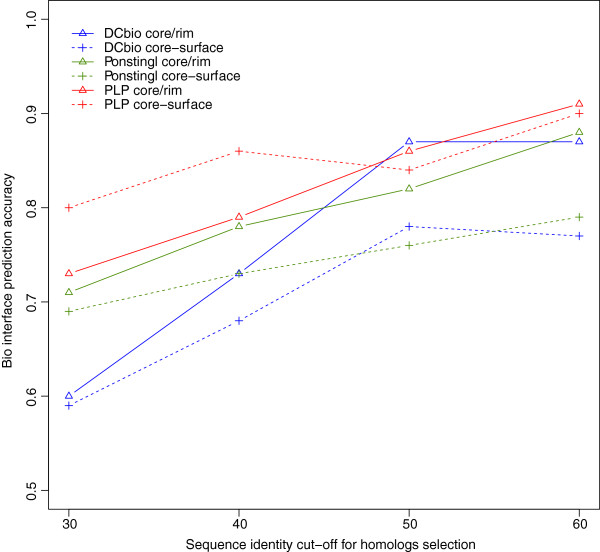
**Our prediction accuracies on biological interfaces versus identity cut-offs used for homolog selection.** The prediction accuracies of our 2 evolutionary methods (core-rim entropy ratio with solid lines and core-surface entropy score with dashed lines) is plotted against different identity cut-offs for selection of homologs to be included in the alignments. For all datasets accuracies are lower when more distant homologs are used in the alignments.

In order to see how the choice of identity cut-off affects our interface predictions we studied the accuracies of predictions with variable identity cut-offs. The results are presented in Figure 5. As we lower the sequence identity cut-off for inclusion of homologs in our alignments the accuracy of the evolutionary predictions clearly degrades. The behavior was similar across different sets of biological interfaces datasets (DCbio, Ponstingl dimers and PLP enzymes). We achieved optimal results with a combination of 60% soft identity cut-off and 50% hard identity cut-off (see Methods).

Choosing a more stringent cut-off is only possible thanks to the size that sequence databases have reached in the last few years. As the growth will only continue in the foreseeable future we believe that our conservative approach will continue giving the best signal to noise ratio in measuring differential selection pressure of interfaces.

### Core versus surface scores

One of the earliest attempts to use evolution to predict biological interfaces [7] compared average sequence entropies of interface residues versus those of the other surface residues. As discussed in the Introduction, this approach was hampered by bias caused by patches of low-entropy surface residues corresponding for instance to binding sites or external active sites. In the search for an additional evolutionary prediction criterion, we took inspiration from that early attempt and introduced an approach comparing the average sequence entropies of interface core residues and of surface residues. In order to reduce bias in the calculations, we employ random pooling of surface residues. Given an interface with N core residues, we sample random pools of N surface residues so that we then can compare the entropy of the core residues versus that of the distribution of surface samples. We then give the final score as the distance of the average core entropy to the mean of the surface samples in units of their standard deviation, in a Z-score-like approach.

Core-surface scores provide a measure of the selection pressure acting on the key residues of an interface compared to a surface “baseline” estimated from the randomly pooled surface residues. In order to further reduce bias, only those surface residues that are involved in none of the interfaces found in the crystal are used for pooling.

Valdar and Thornton [17] did also employ a surface sampling approach in analyzing a limited set of homodimer interfaces, though in their case the statistical significance of the interface versus surface conservation was assessed *via* P-values. Later, Caffrey *et al.*[35] followed that approach and used a Z-test for significance estimation, but concluded that the measured evolutionary signal was not sufficient to predict interface patches from conservation information alone.

### Combining information from the different criteria

As described above we employ three different indicators of the interface character: a geometric one and two evolutionary, core versus rim entropy ratio and core versus surface entropy score. To offer a final prediction of interface character we set out to combine the different indicators into a single call. We decided for a simple majority voting system, where we place more confidence on the evolutionary calls (see Methods). In the case that not enough suitable homologs are available for a certain protein structure, making it impossible to employ the evolution-based criteria, the final call is based on geometry only. In addition we employed the results from the compilation of the DCxtal contact dataset (see Methods for details) to establish hard limits for biological or crystal contact character: areas above 2200 Å^2^ are always considered biological, while areas below 400 Å^2^ are always considered crystal, irrespective of the other indicators. The low hard area limit criterium refers to non-induced [36] protein-protein interfaces, and does not apply to protein-peptide ones.

### Engineering artifacts in the PDB: a word of caution

A further novelty we introduced in our interface classifier method is that of checking for engineering artifacts in the structure being analyzed. This important aspect is, to our knowledge, mostly neglected by computational methods attempting to classify crystal interfaces. In order to produce, characterize and crystallize proteins, structural biologists often need to introduce modifications into their wild-type sequences. These range from point mutations to insertion of affinity tags or to total chimeric constructs. We deal with this issue by first of all finding a reference UniProt sequence for the given PDB sequence. Multiple UniProt assignments to a single PDB entry usually indicate a chimeric construct (*e.g.* the recent structure of the channelrhodopsin light-gated cation channel [37][PDB:3ug9]. In that case, no evolutionary prediction is run and interface classification relies on core size only. If a reasonable reference UniProt alignment exists (as defined by sequence identity and coverage thresholds) then we attempt to predict the interface with all three criteria. In these cases we further check whether the core and rim residues to be scored locate in a region that aligns properly to the reference. Warnings are produced for mismatches; if the number of mismatches exceeds a threshold, again no evolutionary prediction is carried out and the final call is geometry-based.

### Parameter optimization and performance

Several parameters are used in classifying an interface as biological or crystal. Especially important are the cut-offs used for each of the scores: core size (geometric indicator), core versus rim entropy ratio and core versus surface entropy score. In order to optimize those we used our manually annotated DCxtal and DCbio datasets, which contain entries with experimentally verified quaternary structure assignment and with areas in the difficult range 1000–2000 Å^2^. The optimization process with these datasets led to the following cut-off values: 6 core residues for geometry, 0.75 for entropy core/rim ratio and −1.0 for core versus surface scores.

Finally, in order to benchmark our method with a separate independent set we used the well-known Ponstingl 2003 [18] sets of monomers and dimers which we minimally modified (see Methods). This dataset has the advantage of having been employed several times as benchmark in the literature [9,12,38]. In the case of PISA [36] it was also used as optimization set.

In Table 2 we present the results of the optimization and benchmarking steps and for reference we include the PISA performance on the same sets (see Methods for details on how the PISA performance was measured). The three different methods are first assessed separately and then as a single combined predictor. Additionally to the two datasets DC and Ponstingl we also include the statistics for the Bahadur set (a superset of Ponstingl’s) for completeness. Overall, the performance of our final combined predictor compares favorably to that of the PISA server in the 3 sets. The geometric predictor alone is able to classify the interfaces with high accuracy and is helped by the evolutionary ones to further improve the performance in the final call. It must be noted that the evolutionary predictors are in themselves very powerful at classifying interfaces, with sensitivity/specificity figures ranging from 64% to 87%. These figures are not directly comparable to those of the geometric predictor or the combined predictor as they are based on the subset of entries that could be predicted at all (prerequisites are that at least 10 homologs are available and that enough core/rim/surface residues exist). In the analysis of wrong evolutionary predictions we often found cases with problematic alignments, *e.g.* with inhomogenous sequence identity distribution of homologs. Viral or archaeal proteins seem particularly prone to this kind of problem. We are convinced that better filtering and selection criteria will help in further improving the performance of the evolutionary predictors.

**Table 2:** Classification statistics

***EPPIC (based on UniProt 2012_10)***												
	**# entries**		**Geometry**		**Entropy core-rim**		**Entropy core-surface**		**Combined**			
	**Bio**	**Xtal**	**Sens.**	**Spec.**	**Sens.**	**Spec.**	**Sens.**	**Spec.**	**Acc.**	**Sens.**	**Spec.**	**MCC**
**DC** (optimization)	83	82	0.80	0.73	0.82(68)	0.66(64)	0.87(69)	0.76(67)	**0.81**	**0.88**	**0.73**	**0.62**
**Ponstingl** (benchmarking)	88	52	0.85	0.92	0.84(76)	0.66(29)	0.85(75)	0.79(29)	**0.89**	**0.90**	**0.87**	**0.76**
**Bahadur** (benchmarking)	121	185	0.88	0.88	0.82(103)	0.64(114)	0.86(104)	0.77(114)	**0.86**	**0.89**	**0.84**	**0.72**
***PISA***												
	**Acc.**	**Sens.**	**Spec.**	**MCC**								
**DC** (optimization)	0.79	0.95	0.63	0.62								
**Ponstingl** (benchmarking)	0.84	0.89	0.77	0.66								
**Bahadur** (benchmarking)	0.77	0.89	0.69	0.57								

Classification statistics for our own compiled datasets ("DC"), composed of DCxtal (crystal interfaces) and DCbio (biological interfaces), for the Ponstingl 2003 dataset of monomers (crystal interfaces) and dimers (biological interfaces) and for the Bahadur datasets (monomer and dimers). We first present the statistics for each of our indicators separately and the statistics for the combined predictor. PISA statistics compiled by us are shown in a separate table. Statistics are given in terms of sensitivity or rate of correct biological interface predictions and specificity or rate of correct crystal interface predictions. The statistics for the evolutionary methods are based on the total number of interfaces that could be predicted (enough homologs and enough core/rim/surface residues). The numbers for each case are indicated in parentheses together with the corresponding sensitivity or specificity. As well as accuracy values we present the Matthews correlation coefficient (MCC) which gives a better assessment of the predictions in cases where the positive and negative sets are unbalanced (as is the case with the Ponstingl sets). All EPPIC evolutionary predictions are based on UniProt release 2012_10.

### Performance with sequence data growth

**Figure 6:**
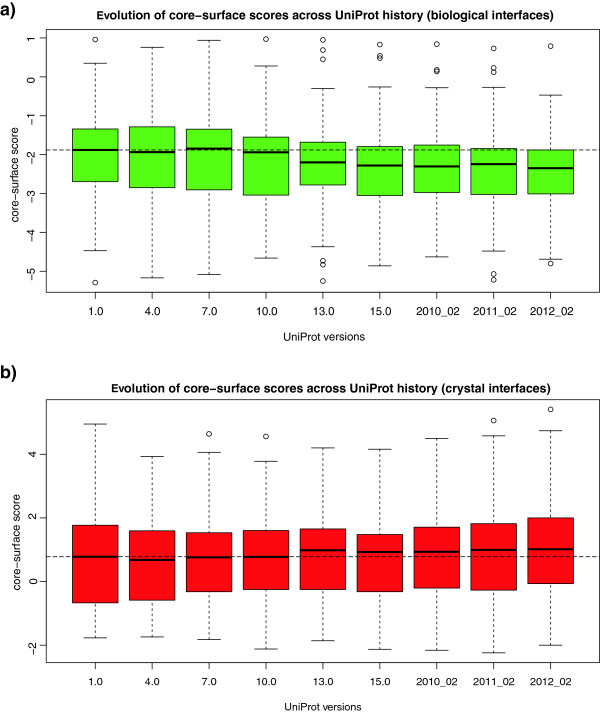
**Core-surface score variation across UniProt history.** The core-surface scores improve on average as more sequence data has become available. Plotted are core-surface scores of **a)** biological interfaces (from DCbio, Ponstingl Dimer and PLP datasets) and **b)** crystal interfaces (from DCxtal and Ponstingl Monomer datasets). The lower the score the stronger the indication of biological interface (our cut-off for classifying bio/crystal is set at −1). The median score for UniProt version 1.0 (2003) is denoted by a dashed line. The chosen versions are separated in time by approximately one year.

In order to more precisely assess the performance of our method we studied the behavior of the evolutionary predictions with the change in sequence data over the last years. The UniProt database has seen an exponential growth aided mainly by an improvement in sequencing technologies that even outperforms Moore's law [39]. We studied the performance dependence of our interface evolutionary predictions with the growth of sequence databases by using archived UniProt versions from the first release appeared in December 2003 to the current one almost 10 years later. The first and more important effect that we observed is a dramatic increase in prediction coverage as the UniProt database grows. For the Ponstingl datasets, coverage rose from 27% in 2003 to 65% in 2012. We are able to predict a particular entry whenever we can find at least 10 non-redundant sequence homologs within 50% identity of the query. Additionally we tried to assess whether the accuracy of the scores increases as alignments get enriched with more sequence data. We thus studied the evolution of the core-surface scores in biological interfaces from a few datasets (DCbio, Ponstingl dimers and PLP enzymes), plotted in Figure 6a. The score distributions across all interfaces exhibit a downwards trend both in terms of median scores and of their spread. Contrastingly Figure 6b present the scores across time for crystal interfaces (DCxtal and Ponstingl monomers), where a slight upwards trend can be observed and not much variation in the spread.

### Web server

**Figure 7:**
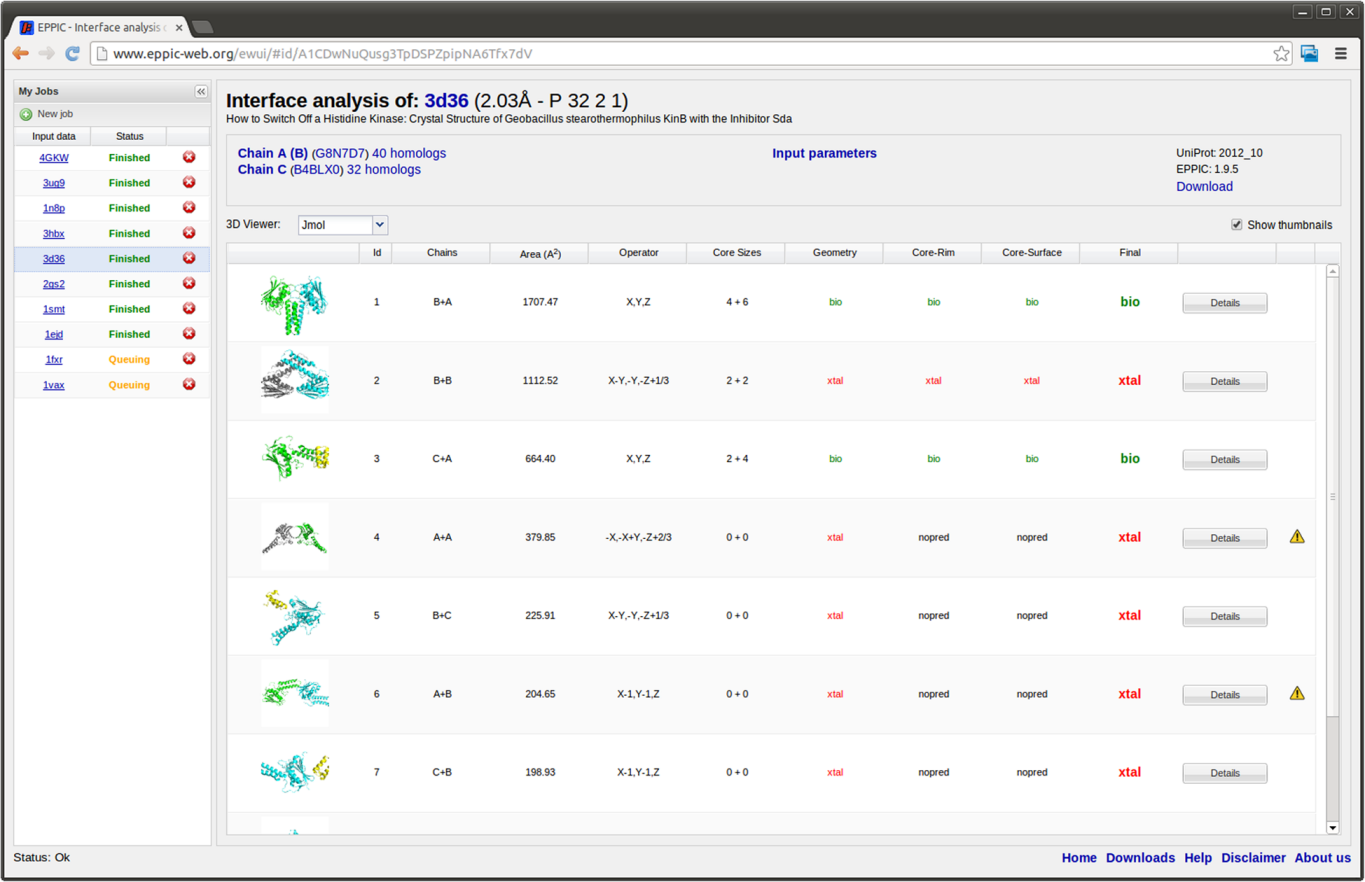
Typical output display of the EPPIC server.

In order to make the EPPIC approach easily accessible to the structural biology and bioinformatics community, we built a web server (http://www.eppic-web.org), with a front-end design centered on clarity and usability. To that end we created a rich interactive web application, based on the Ext-GWT (http://www.sencha.com/products/extgwt) framework. As a minimum input, the user has simply to provide the PDB code of the entry to be analyzed or to upload a coordinate file in PDB or mmCIF format. The user can also access an “Advanced” input panel that allows for changing the parameters for homolog selection and alignment. A collapsible panel on the left provides an overview of the currently running and of the completed jobs. The results page (Figure 7) consists of a top panel, showing the key information about the job and of a dynamic table listing all interfaces present in the crystal lattice. Each row of the table corresponds to an interface, represented as a clickable cartoon-style thumbnail, and shows additional information about the interface. The last columns give the prediction calls (bio or xtal) for all three approaches (geometry, entropy core-rim ratios, entropy core-surface scores) and the final combined call. As an optional extra column, warnings are shown if the residues involved in the interface do not properly align to the reference UniProt entry or other kinds of issues are found in the interface geometry. By clicking on an interface thumbnail, the user can access a 3D view of the interface itself, either through JMol [40] (browser-based, no need for an installed viewer), a local molecular viewer (PDB file) or a PyMOL [41] pse session file.

### A practical example

**Figure 8:**
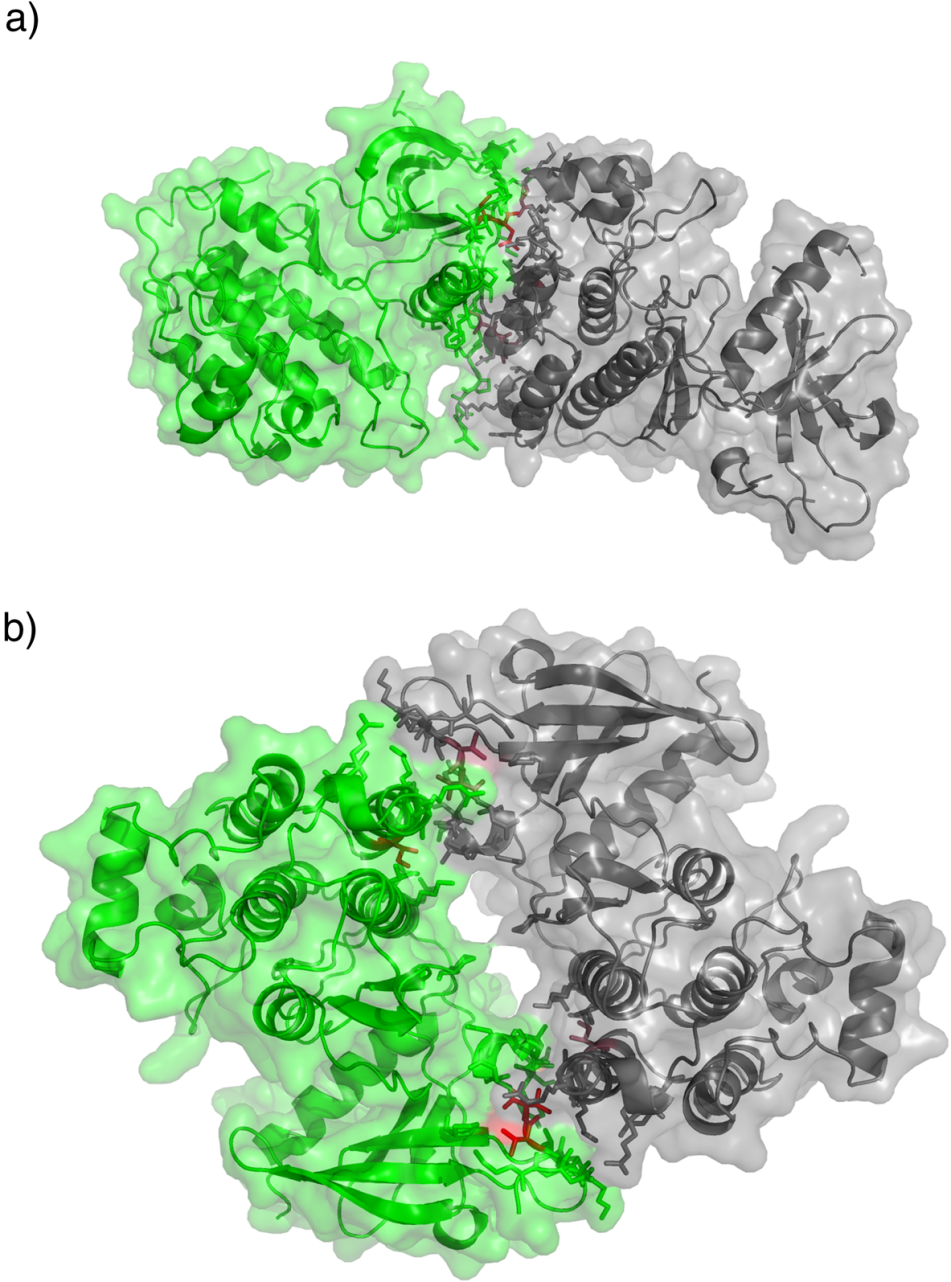
**Identifying the biologically relevant interface of the EGFR kinase.** Asymmetric (top) and symmetric (bottom) dimers in the structure of the epidermal growth factor receptor kinase ([PDB:2GS2]). The two interfaces appear as in the respective PyMOL pse sessions downloadable from the EPPIC web front-end by clicking on interface thumbnails (surface rendering was added for clarity).

An example of using EPPIC in the context of an important structure biology problem is provided by the work of Zhang *et al.*[42] on the mechanism of activation of epidermal growth factor receptor (EGFR), which is based on dimerization. The authors determined the crystal structure of the EGFR kinase domain ([PDB:2GS2]), where either a symmetric or asymmetric dimer, of very similar size (950 and 990 A^2^, respectively), can be chosen as the biologically relevant entity mediating activation. A symmetric dimer, already determined by Stamos *et al*. [43] in a different crystal form, was computationally analyzed by Landau *et al*. [44], who proposed it, among six possible dimer choices, as the key contact controlling inactivation of the receptor. Zhang *et al.* settled the issue with a series of mutagenesis experiments that identified the asymmetric dimer as the relevant one. EPPIC analysis of entry [PDB:2GS2] clearly indicates the Zhang asymmetric dimer as biologically relevant and the symmetric one as a crystal contact. It does so based on clear signals by the entropy core-rim and core-surface criteria, which lead to a correct call for this difficult case in which both interfaces exhibit similar geometrical features (similar number of core residues). Strictly speaking, the asymmetric dimer is unviable since such heterologous interfaces can extend to infinite fibers [45]. Zhang *et al*. do acknowledge this issue and attribute the apparent contradiction to the fact that the crystallized construct is only an intracellular fragment of the full length membrane protein. The symmetric and asymmetric dimers of [PDB:2GS2] are shown in Figure 8 (panels a) and b), respectively) as they would appear to the user in the respective PyMol pse session files provided by the EPPIC web front-end.

## Conclusions

We present here a new, highly effective and easy-to-use method addressing an important issue in structural biology and bioinformatics: that of distinguishing crystal contacts from biologically relevant interfaces. The importance and spread of this problem is now widely recognized: as an effective method to solve it, EPPIC will significantly help in the interpretation of crystal structures, in guiding biochemical experiments on protein-protein interfaces and hybrid approaches in which single components solved by crystallography are to be assembled into large supramolecular entities. Two important conclusions can be drawn from this study: first, that fully buried residues are a key determinant of biological protein-protein interfaces; second, that a stringent sequence selection for the multiple sequence alignments used to measure the evolutionary signal provides a more robust and less noisy way to detect the footprint of evolution in interfaces. This is especially important as the incessant growth of sequence databases fueled by new high-throughput technologies will only increase the usefulness of evolution-based methods. EPPIC bears significant potential for further developments, the most straightforward one being automatic inference of quaternary structure assemblies from the interface predictions, thus providing a complete pipeline from crystal structures to putative biological assemblies. The method is applicable to many problems in both structural biology and structural bioinformatics, to name just a few: validation of structures of oligomeric proteins and of protein complexes, detection of crystal contacts in which one of two partners mimics a biological partner, prediction of protein-protein binding sites in the absence of the structure of a complex and the validation of models of complexes and oligomers.

## Methods

### Compilation and annotation of new reference datasets

In order to compile our monomer dataset (DCxtal) we first gathered a subset of PDB entries by using the advanced query feature of the RCSB PDB site (http://www.pdb.org) on the 21st of December 2010 with following parameters: 1 chain in the biological unit (biounit), resolution better than 1.8 Å, Rfree below 30%, Rsym below 10% and with a sequence redundancy filter at 90% identity. We then calculated all possible interfaces for those entries, taking for our further manual curation those with an interface area above 1000 Å^2^. A further quality control eliminated those entries that generated more than 5 clashes (atoms within 1.5 Å) between chains during the interface calculation process. With this procedure we aimed at finding all putative large crystal interfaces from crystal structures of good crystallographic quality in the PDB. This filtering resulted in a set of 378 PDB entries, which we manually curated by looking into their main references and other literature when necessary. We only took an entry as a candidate for our DCxtal dataset when clear experimental evidence for monomeric state was found in the literature, usually provided by size exclusion chromatography, analytical ultracentrifugation or light scattering techniques [31]. We discarded entries with dubious features or experimental evidence: for instance, putative domain swaps (by visual inspection), debated oligomeric state with conflicting experimental data in the literature or cases where experimental evidence referred to a different fragment than the crystallized construct.

For the DCbio dataset we first took entries that in the above procedure were found to be clearly experimentally verified to be multimeric (thus mostly annotation errors in the PDB as we initially selected entries with 1 chain in the biounit). Then we added 10 PLP enzymes with biological interfaces with areas below 2000 Å^2^. PLP enzymes are known to exist always as dimeric or higher oligomeric assemblies [46]. Finally we proceeded with a similar methodology as above by filtering the PDB for good quality structures with 2 chains in the biounit, aiming to find putative dimers. The interfaces for them were calculated and those with areas between 900 and 1400 Å^2^ were chosen for manual curation by literature search as above.

### Hard area limits

In the above annotation effort, 41 entries contained extremely large (>2000 Å^2^) putative crystal interfaces, from which we could only validate one real monomer ([PDB:1LF2], with an area of 2171 Å^2^). All others were either errors in the biological unit annotations or dubious cases. On this basis we set a hard area cut-off of 2200 Å^2^, above which interfaces are directly called biological without considering the other indicators. We could count only 130 other putative monomers in the PDB (December 2010) having their largest interface area above 2200 Å^2^, resolution <3.0 Å, Rfree<35% and fewer than 5 clashes. Thus, our sample of 41 manually curated monomers represents about a quarter of all putative large monomer interfaces with reasonable quality in the PDB, so the chosen hard cut-off can be considered significant.

### Interface calculation and geometry criterion

We calculated the interfaces for a given entry by applying all symmetry operators corresponding to the entry's space group and finding any pair of chains that had at least 1 atom of each side within a distance of 6 Å. We implemented the interface calculation in Java and integrated it in our code. For surface calculations, we used the implementation of the Shrake and Rupley algorithm [47] written by Bosco Ho (http://boscoh.com/protein/asapy) which we ported into Java. A ball radius of 1.4 Å was used to calculate the Accessible Surface Areas (ASA). Surface residues were considered those exposing more than 5 Å^2^ of their surface. We computed both the ASA of complexed and uncomplexed subunits, finding by subtraction the Buried Surface Area value (BSA):

$$\mathrm{BSA}=\frac{1}{2}\left(\mathrm{AS}A_{\mathrm{uncomplexed}}-\mathrm{AS}A_{\mathrm{complexed}}\right)$$

Surface residues with BSA>0 constituted the interface. We then followed Schärer’s [15] definition to assign the core residues as those with BSA/ASA_uncomplexed_>0.95. Interfaces with more than 6 core residues were considered biological. This value was found in an optimization procedure carried out on the DCxtal and DCbio datasets that maximised both sensitivity and specificity.

### Evolutionary scoring

To calculate sequence entropies for each of the residues of a given PDB structure we used the following procedure: 1) We found the reference UniProt identifier for the PDB sequence by using SIFTS (http://www.ebi.ac.uk/pdbe/docs/sifts) or blasting, in order to control for possible engineering performed on the PDB sequence. 2) Using the reference UniProt sequence we searched the UniRef100 (http://www.ebi.ac.uk/uniref/) database through BLAST [48] to find putative homologs. Only the matching PDB subsequence of the UniProt reference was used for the BLAST search. 3) We then applied sequence identity (soft cut-off of 60% identity, relaxing in 5% steps down to 50% identity until at least 10 homologs were found) and coverage (80%) filters and a hard maximum number of sequences of 100. 4) We then clustered the sequences by using BLASTCLUST [48] and choosing a single representative from each cluster. We did this in an iterative way by starting with a 98% identity clusters and reducing stepwise this threshold if more sequences needed to be eliminated to reach the hard maximum of 100 sequences 5) We finally used the CLUSTALO [49] program to perform a multiple sequence alignment of the selected homologs. 6) Based on that sequence alignment sequence entropies were calculated. The Shannon entropy of an alignment column *i* is given by:

$$s\left(i\right)=-\sum_{k}p_{i}\left(k\right)\log\left(p_{i}\left(k\right)\right)$$

where *p*_*i*_(*k*) is the probability of a residue of class *k* being at position *i* of the alignment. We used a reduced amino-acid alphabet with 10 amino acid classes as proposed by Murphy *et al.*[50].

Entropy values were finally mapped back to the PDB sequences, so that we could compute from those core versus rim ratios and core versus surface scores as described above. For entropy scoring the core residues were chosen with a more relaxed criterium of 70% burial (assigning the remaining interface residues as rim) in order to achieve more statistically significant comparisons. Only if more than 8 of them exist above 70% burial, we make an evolutionary prediction. For the core versus surface scores calculation we drew 10000 samples of N residues (N being the number of core residues in the analysed interface) from surface residues belonging to none of the interfaces found in the crystal.

### Combined predictor

The combined predictor is based on a simple consensus vote from the 3 methods: geometry, entropy core over rim ratio and entropy core versus surface scores. The majority vote (2 out of 3) of the separate calls gives the final prediction. If an evolutionary prediction cannot be made, due to lack of enough homologs or to an insufficient number of core residues, then the final call is the geometric one. In some cases one of the two evolutionary measures fails. For instance core over rim ratio can fail if too many of the rim residues are mutated, or the core-surface prediction can fail if the surface from which to draw residues is too small. In such cases if the geometry and evolution call do not agree, preference is given to the evolutionary one. Additionally hard area limits are used as described above. Special cases like interfaces with disulfide bridges in wild-type residues are treated as biological, disregarding the other indicators.

### Optimization and benchmarking

We optimized the different parameters against the DCxtal and DCbio datasets. A pooled dataset using both sets of biological and crystal contacts was created and used for the runs. A True Positive was counted when our classifier was able to assign a biological interface as biological, True Negative when it could assign a crystal interface as crystal. We ran the predictions with several cut-off parameters for each of the three methods and chose the set of parameters that maximised accuracy ((TP+TN)/(P+N)). In the final statistics together with the accuracy we also quote the sensitivity (*i.e.* True Positive Rate or correct bio predictions from all possible biological interfaces) and the specificity value (*i.e.* True Negative Rate or correct xtal predictions from all possible crystal interfaces). PISA predictions were assessed as follows: for each entry in the pooled dataset the PISA interfaces and assemblies were downloaded as xml files (http://www.ebi.ac.uk/msd-srv/prot_int/pi_download.html). The first item in the assemblies list was taken as the PISA prediction. It was then checked whether the interface of interest was in the list of interfaces engaged by the assembly. The prediction for that interface was then assigned as biological. The interface was assigned as crystal if a) the interface of interest was not in the list of engaged interfaces b) no assembly prediction was given. If the PISA prediction fell in the “grey region of complex formation criteria” then it was considered as a failed prediction and not counted as either biological or crystal.

The Ponstingl 2003 dataset used here for benchmarking consists of two subsets: 1) a crystal interfaces set: largest interface from each entry in the Ponstingl monomers set; 2) a biological interfaces set: largest interface in each of the Ponstingl dimers set. We minimally modified the entries from the original version published in 2003 [21] to make sure the set was up to similar standards of accuracy as our own compiled sets. We did manual curation of 10% of its entries, finding in that process a few problems with the crystallographic quality of some entries and in some cases with the experimental oligomeric assignment. The entries that were modified were:

in monomers dataset: removed [PDB:1A8O] and [PDB:2ABX] as they are known to be dimers, removed [PDB:2HEX] that is a debated monomer/decamer, see for instance discussion in Schärer *et al.*[15]

in dimers dataset: entry [PDB:1RFB] (3Å resolution, no Rfree available) was replaced by [PDB:1D9C] (2Å resolution, Rfree 0.27)

Two additional datasets were used: Bahadur’s monomer [22] and dimer datasets [51] in benchmarking and in Figure 1; and the PLP enzymes biological interfaces dataset from Schärer *et al.*[15] in Figures 5 and 6a.

### Sequence data growth benchmarking

For the historical study section we first downloaded selected versions of the UniProt archived data available at ftp://ftp.uniprot.org/pub/databases/uniprot/previous_releases. We chose 9 versions that were distanced by approximately a year from each other and ranged from December 2003 to February 2012: 1.0, 4.0, 7.0, 10.0, 13.0, 15.0, 2010_02, 2011_02 and 2012_02. The interfaces used to study the score variation across time are further selected from the full lists by choosing only those that have a clear progression in the number of non-redundant homologs: between 5 and 15 homologs available in version 1.0 and more than 20 homologs available in version 2012_02.

### Software

The core EPPIC code was written in Java using the OWL Java library for structural bioinformatics (http://www.bioinformatics.org/owl/) and is licensed under the GPL. The source code is available at the Subversion repository https://systemsx02.ethz.ch/svn/crk. All algorithms have been integrated in the Java code, including interface calculation and ASA calculations. Blast and Clustal Omega are the only external tools, which we then interfaced from Java. The UniProt JAPI (http://www.ebi.ac.uk/uniprot/remotingAPI/) is used for retrieving UniProt data. The web server is written in Java using the Ext-GWT framework (http://www.sencha.com/products/extgwt) and uses Hibernate (http://www.hibernate.org/) together with a backend MySQL database system for data persistency. The job scheduling in the computational backend is done through the open source Open Grid Scheduler/Grid Engine (http://gridscheduler.sourceforge.net/) system. A command-line version of the interface classification software is available for download at the web address http://www.eppic-web.org/downloads/eppic.zip. The web server is essentially a Web GUI to the command line program.

All plots were generated with R [52]. The PyMol [41] molecular graphics system was used for creating figures, thumbnails in server and extensively for analysis.

## Abbreviations

EPPIC: Evolutionary Protein Protein Interface Classifier; PDB: Protein Data Bank; ASA: Accessible Surface Area; BSA: Buried Surface Area; DCxtal: Duarte-Capitani crystal interfaces dataset; DCbio: Duarte-Capitani biological interfaces dataset.

## Competing interests

The authors declare no competing interests.

## Authors' contributions

JMD, MAS and GC performed the analysis of the data. JMD and AS developed the software. JMD and GC conceived and designed the study and wrote the manuscript. All authors read and approved the final manuscript.

## Supplementary Material

Additional file 1 **Tables S1 and S2.** Manually curated monomer and oligomer DC datasets, with experimental evidence from the literature. The "area" column refers to the largest interface in the protein crystal. References mostly given as PubMed id numbers linking to abstracts. Experimental evidence abbreviations used: SEC size exclusion chromatography; AUC analytical gel filtration; AUC (SV) analytical ultracentrifugation sedimentation velocity; SLS, DLS, LS (static/dynamic) light scattering; MALS multi-angle light scattering; MALLS multi-angle laser light scattering; CCL chemical cross-linking; FRET fluorescence resonance energy transfer; NMR nuclear magnetic resonance; SAXS small angle x-ray scattering; MS mass spectrometry; native-PAGE native polyacrylamide gel electrophoresis. (PDF 156 kb) Click here for file
