# Supplementary material for: Protein interface classification by evolutionary analysis
Source: BMC Bioinformatics. 2012 Dec 22;13:334. doi: 10.1186/1471-2105-13-334 (PMC3556496; doi:10.1186/1471-2105-13-334)
Supplement: Additional file 1 — Tables S1 and S2. Manually curated monomer and oligomer DC datasets, with experimental evidence from the literature. The "area" column refers to the largest interface in the protein crystal. References mostly given as PubMed id numbers linking to abstracts. Experimental evidence abbreviations used: SEC size exclusion chromatography; AUC analytical gel filtration; AUC (SV) analytical ultracentrifugation sedimentation velocity; SLS, DLS, LS (static/dynamic) light scattering; MALS multi-angle light scattering; MALLS multi-angle laser light scattering; CCL chemical cross-linking; FRET fluorescence resonance energy transfer; NMR nuclear magnetic resonance; SAXS small angle x-ray scattering; MS mass spectrometry; native-PAGE native polyacrylamide gel electrophoresis. (PDF 156 kb) [file 1471-2105-13-334-S1.pdf]

Supplementary tables

Supplementary Table 1: DCxtal dataset

entry	area	resolution	xtal interfaces used	evidence	reference	comments
2q7d	1783.63	1.6	1	SEC, SLS, comparison to 2odt	17616525	Monomer. The paper states exactly that "biophysical methods like gel filtration and static light scattering indicate a monomeric state" but data is not shown. The same protein was solved by SGC 2odt and does not have that interface at all. Note that the HIS tag from both chains is involved in interface, however only in part of the rim.
2gas	1566.9	1.6	1	SEC	16600295	Paper clearly states it is a monomer, they used SEC and show data.
3c8y	1522.73	1.4	1		2173950, 2544883	Iron hydrogenase 1 from *Clostridium pasteurianum*. The review says it is a monomer, quoting this paper, but it's not clear what's the evidence there. Anyway all publications after that seem to assume that it is surely a monomer, e.g. 9836629 which is the primary reference for 1feh, same protein as this one solved before . That one contains also the same interface (but it's also the same crystal form).
3mhz	1516.29	1.7	1	SEC	20672855,9039918,15243628, 17335404, 4966833	The paper and the (extensive) literature on this PA anthrax protein assume that it is a well-known fact that it is a monomer (82KDa). It's cleaved and the 62KDa part heptamerizes to form a pore. First gel filtration in 4966833, established molecular weight of ~ 100 KDa, thus monomer.<br>Entry 1t6b is the same protein complexed with a human cell receptor and also contains a large putative crystal interface.
2wbq	1515.87	1.1	1	SEC	19490124, 15368580	No info in paper (though they do mention they did gel filtration), but in a previous paper (15368580) characterized as monomer by SEC. Homologous to another protein in this list 2og5 (30% id with good structural conservation). The interface in both cases is the same one anyway (even though space groups are different).
3aap	1382.3	1.6	1	SEC	20159467	Monomer. Authors say so in paper, they used SEC but data not shown

2yz1	1378.66	1.4	1	SEC, AUC	18045614	This ligand binding domain (extracellular domain of a membrane protein) seems to be a monomer in solution as shown by SEC (no data shown) and AUC (sedimentation equilibrium, data shown). As further hint the authors note that the interface is antiparallel and thus unlikely to be formed by a transmembrane protein on a single cell surface. The full length including transmembrane domain could be a dimer.
2ipi	1329.37	1.7	1,2	SEC	17395717	Monomer following publication, they did SEC, no data shown. Four chains in the a.u., with two nearly identical large interfaces (B+A)
3cj1	1124.4	1.7	1	native PAGE, SEC, DLS	17910474	Brenda assigns as dimer. 17910474 claims monomer by native PAGE (data shown) SEC and dynamic light scattering (data not shown)
2eyi	1111.83	1.7	1	DLS, SEC	12657793	a-actinin binding domain, monomer, dimerization domain before, scheme
1pp3	1110.48	1.6	1	SEC	DOI: 10.1021/cg800616q	Monomer by SEC
1ynq	1101.76	1.3	1		16242712	Personal communication by XD Li
2w20	1100.67	1.5	1	SEC	18765901	Truncated version of protein, predominantly monomer, full length may be dimer.
3mg1	1099.32	1.6	1	SEC	20368334	Authors say it is a monomer based on SEC, they show data and looks like well calibrated. Homolog (~70% seq id) 1m98 has the same interface and the paper says that it is a dimer based on SEC, but they don't show the data. Thus we call monomer following the first paper as they seem to have good data.
3cu9	1098.4	1.2	1	SEC	19505290	Monomer by SEC, so the authors claim in the paper, without showing data. Originally the entry in the list was the mutant (from the same study) 3d5y but we replaced it by the wild type 3cu9. Both contain the same interface
1n45	1097.6	1.5	1	CCL, FRET	19556236	Forms dimers/oligomers in ER, monomer when it does not have TM helix.
2hlq	1087.36	1.5	1	SEC	17094948 16982201	Seems monomeric following their very detailed paper (16982201) on purification of this protein. They did SEC and show data (construct D 32-131 is the one used for crystallisation).
1s83	1063.55	1.3	1	SEC	19585993	Monomeric, bovine pancreatic trypsin was used as a standard in SDS-PAGE.
3go5	1063.14	1.4	1	SEC	20399190	Monomer claim the authors, by SEC but no data shown.
3fwk	1046.84	1.2	1	SEC	19375431	Authors claim monomer by SEC. They show the data in supplementary material (Fig. S1). Homolog 2wsi (~50% seq id and with very good structural conservation) does not have that interface.
1gpi	1039.54	1.3	1		11743726	Circumstantial evidence: crystal structure is truncated enzyme, additional residues for full length would disrupt interaction.

1lxk	1033.37	1.5	1	SEC	9365	Monomer in Bacillus sp.
2j0p	1017.8	1.7	1	SEC	16943192	Authors claim monomer by SEC. They present the data (Fig. S2 of supplementary) and they really did a very good job: well calibrated, done at different protein and salt concentrations.
1xgk	1017.46	1.4	1	SEC, DSC unfolding	11679757 15537757	Seems to be a monomer. Determined by SEC (11679757), some data given but no plots. Also main publication for this structure 15537757 claims monomer through DSC unfolding. (Note that the 2 names used for the species of this NmrA protein: Emericella nidulans and Aspergillus nidulans refer to the same fungal species, see wikipedia)
3gkj	1014.66	1.6	1	AUC (SV)	19563754	Authors claim monomer by AUC. Previously supposed to be dimer (17989072) by SEC, but authors say that "Previous results with glycosylated proteins have shown anomalous migration behavior on size exclusion chromatography", that's why they did the AUC.
3m66	1009.59	1.6	1		9118945	MTERF3, binds as monomer to DNA, in 16787637 it is stated that it is a monomeric protein
2wsa	1007.3	1.6	1		20036251	Authors state that it is monomeric. Brenda has orthologs characterized by SEC all monomeric.
3hzl	1005.4	1.6	1		17697998	Stated by the authors to exist as monomer in solution.
1w9q	1005.13	1.7	1	NMR	16533050 15698575	Contains not interesting peptidic ligand (chain S). The second paper 15698575 is a full NMR study of the protein's quaternary structure.
3h30	1328.83	1.6	1,2,3		11574463, 17084631	Comparison with 1JWH shows that the dimerization of the CK2 reg. subunit is NOT that observed in 3h30 (heterotetramer, as confirmed by BRENDA). Thus, accepted
2wbf	1323.25	1.6	1	SEC	13679369, 19591843	Fragment of domain SERA5PE of protein SERA5 of Plasmodium Falciparum. Domain SERA5PE is monomer by SEC (13679369)
2j46	1235.11	1.1	1,2	SEC	doi:10.1016/S0167-4838(02)00287-X	Elutes as monomer
3kk8	1233.14	1.7	1	MALS	20139983	Full length forms tetradecamer, kinase domain is monomer (MALS)
1ejd	1227.05	1.6	1	native PAGE	1577165	Characterized as monomer
3gvo	1222.17	1.6	1	SEC,DLS (for homologue, 79%)	19372537 11303521	Mouse Pumilio-2 Puf Domain. High sequence identity with human (~90%) and drosophila (79%). For Drosophila (3h3d) shown to be monomer by SEC and DLS (11303521). The Drosophila structure (3h3d) is a different crystal form and doesn't have the interface. Human homologues are same crystal form as 3gvo with same interface (e.g. 1m8z).

1j96	1218.8	1.3	1	SEC (homologue, 68%)	11514561	Not clear, according to BRENDA in *Rattus norvegicus* monomer by gel filtration (6435601). Seq id is 68%. Primary citation claims monomer in solution (data not shown). Keep
2xov	1199.74	1.7	1		21256137	Transmembrane protease, up and down arrangement, clearly a crystal contact
1so7	1189.06	1.5	1	SEC, disc gel electrophoresis	6735353	Monomer by gel filtration and disc electrophoresis
2cki	1185.89	1.7	1	SEC	16627477	Monomer, assessed by gel filtration
1lqt	1180.32	1.1	1	SEC	12071965	Monomer, assessed by gel filtration
2ow9	1178.46	1.7	1	SEC	17623656, 9790892	Literature found in MEROPS. Same sequence with similar boundaries analyzed by gel-filtration: monomer
2f37	1158.58	1.7	1	SEC	16882997	Ankyrin repeat, monomer in solution, no data shown
2z6o	1158.39	1.6	1	NMR, SLS	19101823	Ubiquitin protein ligase, monomeric as stated in 19101823 by SLS (data not shown) and rotational tumbling correlation time in NMR.
1ueb	1150.1	1.7	1	AUC, LS	15210970	Monomer by AUC and light scattering
3b37	1148.82	1.7	1	SEC	ISSN: 0002-1369 (AGRICULTURAL AND BIOLOGICAL CHEMISTRY, 1988, 52:217)	Monomer by SEC
1wly	1143.41	1.3	1	SEC	15781461	Monomer by SEC (stated in 15781461). However E. coli homolog 1qor (~40%id and very well conserved structurally) has exactly the same interface and they claim it is a dimer in the paper without presenting much experimental evidence.
1lf2	2171.42	1.8	1	SEC, AUC	12454457 17040901	EC 3.4.23.39. Interesting case: dimer in several crystal forms, monomer in solution under the conditions where protein is active, but higher oligomers can form irreversibly. Keep.
2qb5	1790.2	1.8	1	SEC, SLS, comparison to 2odt	17616525	Authors claim monomer in paper from SEC and SLS but data not shown. There's a HIS tag in the N terminal which is involved slightly in the interface. But most of the interface is formed by the C-terminal. Comparing to same structure but different construct 2odt from SGC, that one does not have this interface at all.
1zlq	1678.52	1.8	1	SEC, AUC	7867647 12960164	Was characterized as monomer in earlier publication 7867647 by SEC, data not shown. Then by AUC in this other paper 12960164 (reference of structure 1uiu). Also comparing to same protein 1uiu (first structure solved for this NikA protein), the interface is not present in that one.

2yvw	1522.84	1.8	1	SEC	1577165	SG. There's not much info about this Aquifex aeolicus MurA enzyme, but there are a few very well structurally conserved (~40% seq id, ~1A rmsd) homologs from E. coli, E. cloacae and others. They all seem to be monomers following Brenda. This early paper 1577165 shows evidence for E. cloacae (3kqa) being a monomer by SEC. The various homologs have different crystal forms and none of the others have this interface. Thus monomer.
1d3h	1483.64	1.8	1	SEC	10673429	Authors state in paper that it is a monomer by analytical gel filtration chromatography. This is human DHODH enzyme, the structurally closely related homolog from Lactococus Lactis (1jue) is a homodimer, the dimerization interface of that one is different from this one
2e1v	1653.41	1.8	1	SEC	17383962	Authors state that it is monomeric by gel filtration.
3n5c	1457.8	1.8	1	SAXS, SEC-MALS	20709080	The authors do analysis by SAXS and by SEC-MALS presenting data and a lot of evidence. This seems to be a very clear monomer.
2wbm	1439.53	1.8	1	AUC	19454024	No data shown for sedimentation equilibrium experiment. Authors state "no dimer has been observed for any of the available structures of afSBDS, which show different crystal packings."
3c1d	1427.07	1.8	1	AUC, SEC, CL	18650935	Sedimentation velocity (data shown), homo-bifunctional chemical crosslinking (not shown)
1ndb	1386.57	1.8	1	SEC	12526798	SEC, data not shown. Consistent with publication by Ramsay
2x26	1377.16	1.8	1	SEC	20383006	Interesting interaction: as in fim proteins there is a 13-amino-acid tail (but C-terminal) that folds within the cleft of the next monomer (cloning artifact: coupling of protein to GFP for estimation of expression levels). Protein runs as monomer in SEC (data not shown). No homologs in PDB
3kh7	1333.8	1.8	1		20544959	Most likely monomer, though no data provided. Homolog from E.coli is monomer 11843181, rmsd between 3kh7 (pa) and 2b1k (ec) 0.95 A
1toa	1294.51	1.8	1	SEC	10404217, 10400603	Monomer by SEC
1ffr	1226.41	1.8	1	SEC	Annals of Microbiology, 55 (3) 213-218 (2005)	Chitinase from S. Marcenses (bacterium), uniprot P07254. It's identical in sequence to Sanguibacter C4's chitinase (uniprot Q2V9S9) and that one is monomer by SEC with data (Tao YONG, Jin HONG, Long ZHANGFU, Zhang LI, Ding XIUQIONG, Tao KE, Ge SHAORONG, Liu SHIGUI, Annals of Microbiology, 55 (3) 213-218 (2005), Purification and characterization of an extracellular chitinase produced by bacterium C4) (link to pdf). Brenda also has many monomers for bacterial chitinases
1vqq	1215.7	1.8	1	SEC	8163510	Seems to be monomeric by SEC

1n4g	1162.15	1.8	1	MALLS	20621636	CYP121, it's monomer
3ita	1142.07	1.8	1	native PAGE	19807181	Publication claims that PBP6 behaves as monomer in solution
1woq	1129.82	1.8	1	SEC	12839753	15377666 publication claims monomer, evidence given in 12839753
1cqx	1110.84	1.8	1	SEC	8557026, 218634	Authors claim structure to be monomeric but no evidence given. See 218634 for clear SEC evidence.
2eqa	1105.25	1.8	1	LS	18004774	As described in publication
3mhj	1102.84	1.8	1	SEC	18436240	Human tankyrase 2, most likely monomer by comparison with tankyrase 1 2RF5 (evidence for tankyrase 1 given in 18436240), sequence identity is about 72%
2fgz	1091.03	1.8	1	Homology	16650854	Crystal Structure Analysis of apo pullulanase from Klebsiella pneumoniae, monomer in Klebsiella aerogenes (seq id 85%)
1fpo	1087.78	1.8	1	SEC	9144776	Protein seems larger than monomer, but smaller than dimer. interpreted as non-globular by authors. It makes sense if compared to structure
3lvd	1082.92	1.8	1	SEC	20220148 12693991	Green fluorescent protein mutant (aceGFP-G222E), wt is monomer by SEC; since G222E does not affect interface, accept
3irb	1060.05	1.8	1	SLS, SEC	20944206	PISA seems to predict 2mer or 4mer, experiments seem to tell otherwise, no data shown
1utj	1057.15	1.8	1	SEC	8896331	Trypsin of Salmo, SEC mentioned in publication
3f0o	1043.2	1.8	1	SEC	3542021	Monomer in solution, but crystal interface looks quite real
2h44	1038.64	1.8	1	SEC	16735511	Monomeric fragment (535-860). SEC was done on 508-865, the full-length protein is dimeric
1t8g	1029.16	1.8	1	SEC	15340171	Phage T4 lysozyme mutant L32A/L33A/T34A/C54T/C97A/E108V. Lysozyme is a monomer and the author state that "soluble and monomeric as judged by elusion profiles from sizing columns (data not shown)"
1g6a	1019.59	1.8	1	SEC	11148033, 235307	PSE-4 beta-lactamase, monomer by SEC (the authors quote an early paper where there is a calibrated SEC)
2zyr	1009.28	1.8	1	SEC, active site titration with [3H]DFP, native PAGE	19447113,, 10620337	AFL from *Archeoglobus fulgidus*. Crystallizes also in a form with only one monomer per a.u. (2zys), shares interface with 2zyr. Monomeric as determined by previous biochemical study
3els	1005.04	1.8	1	SEC	19010333	Clear chromatogram with calibration, monomer

Supplementary Table 2: DCbio dataset

entry	area	resolution	assembly	bio interfaces	evidence	reference	comments
2fwv	2085.26	1.7	2	1	DLS, SEC	17172346	It's a dimer, wrongly annotated as monomer. They check it with several methods in the paper: DLS, SEC (showing data for both) and homology. PISA predicts wrongly a tetramer as the most likely assembly
1ytq	1950.11	1.7	2	1	SEC, DLS	17327390	It's a dimer, wrongly annotated as monomer. The abstract says it already and they show it in the paper with SEC and dynamic light scattering.
1v2x	1705.29	1.5	2	1	AUC	15062082	It's a dimer, wrongly annotated as monomer. In paper they state they did AUC but don't show the data. Strangely the BSA they quote in the paper is 1353 A2 instead of our value (or PISA's) ~1700 A2.
1pkh	1673.65	1.4	3	1,2	SEC	12909016	It's a hexamer, wrongly annotated as monomer. Clear from crystal and also proofed with SEC in paper. Actually it's a dimer of trimers. They proof in the paper that it's a hexamer and not a trimer with SEC, but it's not totally clear whether the hexamer is real (could be simply low affinity). As the 100% clear one is the trimer (interfaces 1,2) we'll take those 2 as bio, interface 4 would be the one corresponding to the dimer of trimers oligomerisation.
1r5y	1554.08	1.2	2	1	SEC, non-covalent MS	7665516 19627989	Zymomonas mobilis (this one) was considered a monomer (SEC, data not shown), but a recent paper shows that it is a dimer by non-covalent MS and that it is the dimer that binds one substrate tRNA molecule at a time.
1kq3	1486.08	1.5	4	1,2	SEC,EM,homology	11566129 11134946	A tetramer. No publication (SG). But homolog 1jq5 is a tetramer (clearly stated in paper with support from EM and SEC data). Identity is ~40% but structure is very well conserved. They both contain the same interface and furthermore they both crystallize in same space group (I 4 2 2). Actually there's a tetramer/octamer discussion for 1jq5, in 11566129 they say an octmer fits the EM data but looks not so convincing, in 11134946 theres SEC evidence for octamer, but they also mention an earlier paper which claims tetramer. Taking only interfaces 1,2 (tetramer) and not 3 (the octamer one)
1jq5	1318.52	1.7	4	1,2	SEC,EM	11566129 11134946	See 1kq3 above.
2h7i	1481.31	1.6	4	1,2	SEC	17588773	Tetramer. The protein has been solved many times (26 entries with 100% id in PDB). The paper from 2idz explicitly says it's a homotetramer from SEC data (not showing data).
2nzl	1299.14	1.4	4	1	SEC	17669354	A tetramer. SEC evidence in this paper found in reference of main paper of entry 2W0U (same protein as this one).
1uj6	1281.61	1.7	2	1	SEC,DLC	13679361	Homodimer by SEC and DLC according to main reference of structure
1ju3	1268.31	1.6	2	1	SEC	11742345 20436035	Dimer by SEC (20436035). Main reference and PDB annotation say monomer
1sml	1258.8	1.7	4	1,2	SEC,AUC	9811546	Wrong annotation: tetramer by SEC (older references from main paper) and AUC sedimentation equilibrium with data shown (9811546)
3fah	1240.29	1.7	2	1	AUC,SEC	8354279	Wrong annotation, characterized as dimer.
1lzl	1207.56	1.3	2	1	SEC	12421810	Dimer by SEC
3iue	1150.47	1.7	2	1	SEC	11669627	Dimer by SEC in 11669627

1vk5	1144.85	1.6	2	1	SEC	16511118	"Gel filtration indicates homodimer" (data not shown)
2exb	1899.64	1.8	2	1	AUC	16411754	Dimer, the paper says that "it forms a tightly bound dimer" from AUC analysis (sedimentation and equilibrium), but data not shown.
3a2q	1745.79	1.8	2	1	SEC,AUC	923591	It's a homodimer by SEC and AUC, see 923591
3h6d	1665.4	1.8	3	1			It's a homotrimer. Couldn't find the citation with the exact experimental evidence, but it is assumed by all authors that it is known to be a trimer. There are 10 structures for this protein in the PDB. All showing the trimer, e.g. 1snf which is also a different crystal form
1s2z	1621.4	1.8	2	1	AUC	2835096	It's a homodimer by AUC. Has 2 large (>1500A2) interfaces. Both are in all 10 structures for this protein in PDB, all of them same crystal form though (see protcid)
2z1n	1586.77	1.8	2	1	SEC	18175326	Dimer or tetramer by gel filtration (data not shown). Actually there are 2 different interfaces that form 2 possible dimers and altogether a possible tetramer. The gel filtration gives a ratio 10:1 dimer to tetramer. But it's not known which one of the 2 interfaces is the possible dimer observed. We take only interface 1 as a sure bio to stay in the safe side
2vef	1556.35	1.8	2	1	SEC	3114239	Dimer by gel filtration, see 3114239. Other homologs from similar organisms (e.g. 1eye from M.Tuberculosis) superpose well, have same interface and are known to be dimers, see 11007651
1bs1	1483.09	1.8	2	1	SEC	4921568	Was earlier characterized as homodimer. It's been solved many times and all the structures in the PDB have the same interface
3lw6	1010.95	1.8	2	1	SEC,UDP binding stoichiometry	20236943 19032152	Catalytic domain of Drosophila beta1,4-galactosyltransferase-7 (73% similarity to human). For human enzyme (delta 1-81) gel filtration and UDP binding stoichiometry show it is a dimer. Interestingly this entry (Drosophila) contains quite a long N-terminal expression tag taken from the homologous bovine enzyme. Anyway the tag is not even seen in the density and in any case doesn't come close to the interface.
3f3e	1270.99	1.8	2	1	analogy to serotonin receptor	16041361	Dimer according to 16041361
2vr4	1265.02	1.8	2	1	SEC,DLS	17287210	Dimer by SEC and DLC data not shown, reference is of same protein solved earlier by same group (2je8). Brenda has dimer or higher oligomer for same EC (3.2.1.25) in related organisms
1x7v	1191.21	1.8	2	1	SEC	16049913	"The PA3566 protein crystallizes as a trimer, although the functional unit is most likely a dimer, as are other members of this structural superfamily." confirmed by SEC
1f2d	1892.91	2.0	2	1		15189147	PLP enzyme, known to be dimer or higher oligomer, see review 15189147 by Eliot and Kirsch
1lw4	1857.20	1.9	2	1		15189147	PLP enzyme, known to be dimer or higher oligomer, see review 15189147 by Eliot and Kirsch
1n8p	1969.27	2.6	2	1		15189147	PLP enzyme, known to be dimer or higher oligomer, see review 15189147 by Eliot and Kirsch
1qop	1531.10	1.4	2	1		15189147	PLP enzyme, known to be dimer or higher oligomer, see review 15189147 by Eliot and Kirsch
2aq6	1195.11	1.7	2	1		15189147	PLP enzyme, known to be dimer or higher oligomer, see review 15189147 by Eliot and Kirsch

2bhs	1487.99	2.7	2	1		15189147	PLP enzyme, known to be dimer or higher oligomer, see review 15189147 by Eliot and Kirsch
2cft	944.77	1.8	2	1		15189147	PLP enzyme, known to be dimer or higher oligomer, see review 15189147 by Eliot and Kirsch
2e7j	1668.16	2.4	2	1		15189147	PLP enzyme, known to be dimer or higher oligomer, see review 15189147 by Eliot and Kirsch
2ecq	1623.08	1.9	2	1		15189147	PLP enzyme, known to be dimer or higher oligomer, see review 15189147 by Eliot and Kirsch
2rkb	1104.54	2.8	2	1		15189147	PLP enzyme, known to be dimer or higher oligomer, see review 15189147 by Eliot and Kirsch
1eej	856.37	1.90	2	1	SEC	7536035	Well characterized as homodimer, see 7536035.
1o17	933.97	2.05	2	1,2	SEC	11298741	Well characterized as homodimer, see 11298741. Two copies of the interface in the ASU.
1ze3	1473.17<br>1093.65	1.84	3	1,2	SEC	15920478	Ternary complex of FimD-C-H. Characterized as complex by SEC
3d36	665	2.03	4	1,3	Mutagenesis	19101565	Well characterized complex (on interface 3) of KinB (chains A,B) and small protein Sda (chain C). Interface 1 is for homodimer A+B. Interface 2 is really interesting because it is a crystal contact that replaces the bio contact of interface 3 in the other (symmetric) side of the molecule. The paper is very thorough and they check the interface with different techniques, including mutagenesis.
3r0n	901.84	1.3	2	1	SEC,Native PAGE	22547693	Immunoglobulin variable domain of Nectin-2. The reference is the primary citation of same protein 4dfh
3da8	911.22	1.3	2	1	DLS,SEC	19394344	Authors did both DLS and SEC but show no data, they solved 2 structures for the same protein in different crystal forms, both have the interface providing further evidence.
2c4w	914.5	1.6	12	1,2	AUC	1554351	Type II DHQase from H. Pylori. It's a dodecamer by similarity to 2y71 (M. Tuberculosis, only 30% seq id but amazing structural conservation at the dodecamer level, pymol aligns the 2 dodecamers downloaded from PISA with 1.4 rmsd) and to 1gu0 (35%id, very good structural conservation at dodecameric level). 2y71 and 1gu0 have been well characterized biophysically (1554351) as dodecamers. Interfaces 1 and 2 are engaged to form the dodecamer.
3jrz	933.25	1.7	2	1	NMR	19959472	They solved both the crystal and the NMR. In the abstract they say that it is a dimer in solution, but the only proof of it in the paper is from the NMR NOE peaks that are assigned to inter/intra molecular ones via the X-ray structure. Additional clues: 1) 2 crystal forms solved have the same interface, 2) a well conserved homolog of E coli 3hpw (~40%id) also has the same interface
3f6q	949.99	1.6	2	1	SEC	19074270	Citing the paper: "the complex remained intact through further rounds of ion-exchange and size-exclusion chromatography". They also tried mutagenesis on several residues, especially mutant F42A caused near complete loss of binding. By the way, the F42 is the only core residue in that side of the interface
2wxd	960.02	1.6	2	1	SEC,AUC	2369130<br>9195886	Well charaterized dimer, original reference is 2369130. Solved a few times in the PDB.
2bz6	981	1.6				16621574	FACTOR VIIA heavy and light chain linked by S-S bridge. The original single chain is cleaved by other factors of the extrinsic blood coagulation pathway into the heavy and light chain (P70375 UniProt)

2d0d	1003.06	1.7	2	1	SEC	16233251	Homodimer by SEC. This is a mutant but I couldn't find the WT, there are a few other mutants in PDB. In any case the mutation is not in interface.
3cm3	1012.27	1.3	2	1	AUC-SV	19211553	Homodimer by AUC - sedimentation velocity, they show data.
3o1n	1017.98	1	2	1	SEC	21291284 8216229	AroD from *Salmonella enterica serovar typhimurium* ; AroD from *Salmonella typhi*, 100% id, is dimer by SEC (8216229)
3h0n	1018.4	1.5	2	1	SEC,SLS	20944211	SG, new fold (ABATE domain), published.
2v52	1030.07	1.5	2	1	fluorescence anisotropy	19008859	MAL-RPEL2, Kd by fluorescence anisotropy 0.1 uM.
1zlh	1033.56	1.7	2	1	inhibition kinetics, titration	15961103 15561703	Heterocomplex carboxypeptidase A1:proteinaceous inhibitor. It binds 1:1 (titration) with Ki ~ 1nM
2dvn	1045.17	1.6	2	1	DLS	18062990	
3ovp	1060.91	1.7	2	1	SEC, AUC	20923965	
2i7d	1077.94	1.2	2	1	SEC	2157703	The main reference for this entry is 17985935
3bzl	1085.28	1.2	2	1	Dimer because the two chains originate from a self-cleaved protein which retains its fold	18451864	Special case: this C-terminal domain of the EscU protein from the type III secretion system of Gram-negative bacteria self-cleaves into two polypeptides and the two chains stay together. Originally the entry we had from the filtering was 3bzy, but it is a mutant, wt is 3BZL (1.71 A).
2car	1099.46	1.1	2	1	SEC	17138556<br><br>11278832	Clear dimer by SEC, they show the calibration curve
3gus	1221.37	1.5	2	1		16597834 16399376	Very well studied human glutathione S-transferase enzyme (2.5.1.18) type p1, standard name hGSTP1-1. Known to be homodimeric, could not find the exact reference. Many structures in PDB, all having the interface.
3jyo	1224.52	1	2	1	SEC	18566515	Homodimer by SEC
1uz3	1228.26	1.1	2	1	SEC,AUC	15978617	N-terminal domain of EMSY protein (Q7Z589), homodimer in solution by SEC, AUC and even yeast two-hybrid evidence. Kd established at ~2uM
2wtm	1243.68	1.6	2	1	DLS	20058325	Homodimer in solution by DLS. They solved 2 different crystal forms of the protein, both containing the interface.
2a5l	1252.84	1.7	2	1	AUC,SEC	16322580 9694845	Dimer-tetramer equilibrium, we take thus the 1st interface as valid (corresponding hopefully to dimer). Another structure within 95% id has been solved: 1zwl. Both have in common interfaces 1 and 2 and are 2 different crystal forms.
2y39	1294.51	1.4	2	1	SEC,AUC,NMR	18825506	A homodimer by SEC, AUC and NMR. 3epv is the same protein with same interface except that it is slightly bigger, maybe because of different bound metal? In any case the evidence looks quite solid.
2ab0	1296.42	1.1	2	1	DLS	16181642	Protein YajL from E coli, dimer by DLS and homology to structurally very close human protein DJ-1 (1p5f)
3epw	1302.63	1.3	2	1	SEC	11292348	Homodimer by SEC. Solved a few times in PDB. Reference is from first one 1hoz, which lacks part of a helix in the interface (missing density I guess), resulting in a much smaller area for the interface.
2g2u	1304.7	1.6	2	1		21294157 16809340	Well studied complex of SHV-1 + BLIP. Kds have been measured for WT (2uM) and mutants. We had originally the mutant 3n4i and I have replaced it by the WT 2g2u.

2vvt	1340.2	1.7	2	1	MALS,AUC	17568739	Homodimer in solution, from MALS and AUC. They did a thorough study on this and many related homologs from other bacteria, characterizing and crystallizing all of them. Seems very clear
1p5f	1343.76	1.1	2	1	SEC	12855764	Well characterized human DJ-1 protein. We have a homolog in this list (2ab0). They did SEC and show data. We originally had a mutant in the list (2rk3), I've replaced it by WT 1p5f at 1.1A resolution
3itf	1359.9	1.5	2	1	SEC,SGS	21239493	Homodimer by SEC and Sucrose Gradient Sedimentation, through them they determine a mass of 31KDa. All data shown
3kd2	1371.5	1.8	2	1	SEC,AUC-SV	20118260	Homodimer by SEC, AUC-SV, showing data, looks solid. Originally we had mutant 3kda in the list, replaced by WT 3kd2, resolution 1.8
2w6a	1394.15	1.4	2	1	MALLS, AUC-SE	19136011	Clear case
2y27	1395.2	1.6	2	1	SEC	21388965	Clear case
